# Visual-Haptic Size Estimation in Peripersonal Space

**DOI:** 10.3389/fnbot.2020.00018

**Published:** 2020-04-16

**Authors:** Nikolaos Katzakis, Lihan Chen, Frank Steinicke

**Affiliations:** ^1^Human-Computer Interaction, Department of Informatics, Universität Hamburg, Hamburg, Germany; ^2^School of Psychological and Cognitive Sciences and Beijing Key Laboratory of Behavior and Mental Health, Peking University, Beijing, China

**Keywords:** visual, haptic, size, force-feedback, perceptual estimation, peripersonal space, virtual reality

## Abstract

In perceptual psychology, estimations of visual depth and size under different spatial layouts have been extensively studied. However, research evidence in virtual environments (VE) is relatively lacking. The emergence of human-computer interaction (HCI) and virtual reality (VR) has raised the question of how human operators perform actions based on the estimation of visual properties in VR, especially when the sensory cues associated with the same object are conflicting. We report on an experiment in which participants compared the size of a visual sphere to a haptic sphere, belonging to the same object in a VE. The sizes from the visual and haptic modalities were either identical or conflicting (with visual size being larger than haptic size, or vice versa). We used three standard haptic references (small, medium, and large sizes) and asked participants to compare the visual sizes with the given reference, by method of constant stimuli. Results show a dominant functional priority of the visual size perception. Moreover, observers demonstrated a central tendency effect: over-estimation for smaller haptic sizes but under-estimation for larger haptic sizes. The results are in-line with previous studies in real environments (RE). We discuss the current findings in the framework of adaptation level theory for haptic size reference. This work provides important implications for the optimal design of human-computer interactions when integrating 3D visual-haptic information in a VE.

## 1. Introduction

During daily operation, haptic inputs (including force feedback) to the human body (e.g., hands), provide a genuine and instant sensory experience for human operators and streamline the intuitive and natural multisensory interaction. During the interaction, sensory information is transmitted and distributed between the sender (the operator) and the receiver (real world). With the recently emergent advances in virtual reality (VR), rich and immersive sensory experiences become possible, such as through our enhanced perception of audiovisual stimuli (Van der Meijden and Schijven, 2009). However, haptic feedback technology is still relatively under-developed in the quest to approximate the genuine sense of “reality.” Moreover, it is still a challenge to touch and manipulate various objects (even with force feedback) in VR as we do in the real world, and psychophysics measurements in this regard are lacking.

To address this problem, sophisticated haptic displays have been designed. A number of those displays (Dataglove, 3DS Touch, http://www.3dsystems.com) offer a convincing haptic sensation in some situations. Stylus-based haptic inputs, externally grounded shape displays (Follmer et al., 2013; Abtahi and Follmer, 2018), wearable (Katzakis et al., 2017), mid-air (McClelland et al., 2017), etc., have an advantage over other solutions in that they do not require the user to carry a heavy device or constantly hold a controller (like a joypad) in their hands. Typically, with the stylus, users can efficiently explore a virtual object through a single point (corresponding to a fingertip).

The potentially wide applications of haptic inputs in VR have been hindered by some practical constraints including higher cost, limited workspace bounds and most importantly, an insufficient understanding of the working principles of crossmodal correspondence between different sensory stimuli and the multisensory integration during the haptic-feedback based operation.

This work targets applications, such as immersive teleoperation (Van der Meijden and Schijven, 2009), in which the operator is wearing a head-mounted display (HMD) and uses the haptic device to teleoperate a robotic arm. The workspace of the haptic device is, from the user's perspective, different than the typical (remote) working space in which the operators reach their arms; It is therefore necessary to transform and map the sensory properties, such as visual sizes and haptic sizes, and this raises questions regarding *gain* between different sensory properties. To this end, demonstrating how humans perceive sizes, especially when they are conflicting from different sensory modalities in peripersonal space, is an important step that must be made in order to understand how virtual objects or remote objects should be displayed/rendered in during teleoperations.

## 2. Related Work

There is a large body of work that has attempted to integrate haptics in Virtual Reality (Stone, 2001). Another body of work in virtual and augmented reality has used vision to guide/manipulate haptic sensations (Punpongsanon et al., 2015; Katzakis et al., 2017) and thus modulate and even modify the passively received haptic sensations. In summary, the interaction between visual stimuli and tactile inputs have been implemented in different application fields (desktop VR vs. walking with an HMD), different platforms (Augmented reality vs. Virtual reality) and different tactile properties (surface vs. stiffness). We detail some examples below.

Kokubun et al. (2014) conducted experiments to explore the effect of visuo-haptic interaction of normal and shearing forces with a rear-touch interface. Their study suggested the effectiveness of the setup: more than 80% of participants perceived greater stiffness with the deformed model than the model without deformation. Ban et al. (2014) proposed a visuo-haptic system to display various shapes which have curvature, edges, and inclined surfaces, by using a simple physical device for transmutation and by exploiting the effect of visuo-haptic interaction. In their study, they built a transmutative device, which the user could easily touch. The device does not undergo significant transformation, but its surface can be slightly modulated to be bumping in and out, and rendered various shapes (with various angles, length, and curvature). Their results suggest that displaying each primitive shape can help to render more complex objects with subtle transformation techniques (Ban et al., 2014).

Lecuyer and Burkhardt (2015) evaluated the influence of the control/display (C/D) ratio on the perception of mass of manipulated objects in virtual environments (VE). In two experiments, they asked the participants to identify the heaviest between two virtual balls. Participants could estimate the weight of each ball through a haptic interface and at the same time look at its synthetic display on the screen. Participants did not know in advance the two parameters between each trial: the difference of mass between the balls as well as the C/D ratio used in the visual display when weighing the comparison ball. They found that the control-display ratio influenced the result of the mass estimation task and sometimes even reversed it. The absence of gravity force largely increased this effect. These results suggest that if the apparent visual motion of a manipulated virtual object is amplified as compared to the motion of the user's limb (i.e., if the C/D ratio used is smaller than 1.0), the user feels that the mass of the object decreases. Thus, decreasing or amplifying the motions of the user in a VE can strongly modify the perception of haptic properties of objects that are being manipulated. In this way, designers of virtual environments could use these results to avoid potential perceptual aberrations when they implement the relevant tasks (Lecuyer and Burkhardt, 2015).

Following up from the work of Yokokohji et al. (1996), with a similar paradigm, Abtahi and Follmer (2018) explored angle redirection, resolution and speed change by modifying the Control-Display ratio. They demonstrated that it is possible to redirect up to 40° and scale up to 1.8 to increase the resolution of shape displays.

Matsumoto et al. (2017) proposed a visual and haptic display system that comprised of a portable passive haptic device and an HMD. They employed visuo-haptic integration to emulate a wide range of perceived stiffnesses while at the same time avoiding mechanical actuators that could make the device bulky and power-consuming. The user sees his or her own rendered hand via an HMD with its finger flexion appropriately modified in relation to presented virtual stiffness. They experimentally verified that the proposed system could display both a pinchable elastic ball and a rigid undeformable one (Matsumoto et al., 2017). The interaction between visual and haptic modalities has also been implemented in augmented reality (AR). In an interactive AR environment, Bianchi et al. (2006) explored the overlay of the computer-generated objects, by providing accurate haptic feedback from real and virtual deformable objects and introducing the landmark occlusion on tracking stability during user interaction.

Recently, Zhao and Follmer (2018) presented an algorithm for haptic retargeting. The work contributes a spatial warping approach that allows users of VR to remap objects of arbitrary shape onto haptic objects. This approach could potentially be used with force feedback, with haptic devices, such as the 3DS Touch family of devices. During the visuo-haptic interaction, there could be multiple semantic mappings. Blanch et al. (2004) designed two semantic metaphors (sizes): one size for motor space targeting the importance of manual manipulation and one size in visual space for the amount of information being given. Importantly, the decoupling between visual and motion size was implemented by changing the C/D ratio as a function of distance of the cursor to nearby targets. By taking advantage of the independent manipulation of motor and visual (widget) sizes, traditional graphic user interfaces (GUIs) have been redesigned.

Visuo-haptic interaction has been recently explored in more ecological scenarios. In addressing the practical difficulties in walking and tracking the surrounding environment by wearing head mounted displays, Nagao et al. (2017) presented “Infinite Stairs,” in which they simulated haptic feedback by providing small bumps (reflecting the edge of the steps in the VE) under the feet of the user, and the visual images of the stairs and shoes. This system has successfully enabled users to experience nearly all kinds of virtual stairs with vivid haptic feedback. The visuo-haptic interaction has been extended in the field of pedagogy. In teaching STEM (Science, Technology, Engineering, and Mathematics), learning about nanotechnology has gained popularity by implementing visuohaptic simulations of point charges and their interactions. Students in visuohaptic (VH) groups were more motivated and developed positive attitude toward learning than their peers in visual-only (V) groups (Park et al., 2010; Rubio, 2012; Rubio et al., 2018; Yen et al., 2018).

Finally, Ban et al. (2013) explored altering the shape of an object with a video-see-through HMD. For all the above cited visuo-haptic interaction studies in VE, to our best knowledge, there is no information about how the visuo-haptic mapping in sizes could be perceived and learned/transferred by using traditional force feedback haptic devices (3DS Touch family of devices). This line of research is important since the exploration of objects' edges and hence the inference of their sizes (including both visual size and haptic size) is common during peripersonal motor actions in our daily life. Moreover, depending on the complexity of the task at hand, users of VR systems could use haptic information to pick up objects with different mean (haptic) sizes when the objects are (partially) occluded. There is a gap in the literature concerning how human operators adapt to and resolve potentially conflicting information between visual size and haptic size and make appropriate perceptual decisions to execute the right action. The present study aims to bridge this gap.

## 3. Experiment

In this section we describe the material and methods used in our study.

### 3.1. Participants

Twenty-five volunteers (age 22–38 years old, *M* = 28.5, 11 females–14 males) participated in the experiment. Most of the participants were students or staff members from the local department. All participants had normal or corrected to normal vision, and they signed an informed consent form before taking part in this experiment. None of the participants suffered from a disorder of equilibrium. The study was approved by the Ethics committee of Hamburg University.

### 3.2. Apparatus

**Figure 1:**
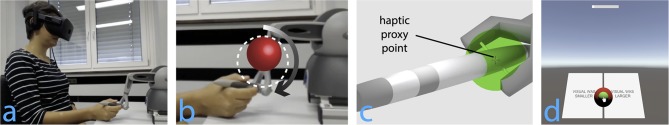
Experiment setup: **(a)** Participants mounted the Oculus Rift during the experiment, in which **(b)** virtual objects were rendered inside the haptic workspace of the haptic device (Geomagic Touch), adjacent to the original location of the haptic device. As illustrated in **(c)** during the homing phase of the task the user (typified as a cursor) was superimposed on the haptic stylus hinge center (haptic proxy point). The view through the head mounted display (HMD) is shown in **(d)** with a progress bar, a green cursor, a visual stimulus in red, and a response UI with hand cursor (for reference).

Participants sat on a height-adjustable chair and desk (Figure 1a). We used the adjustable chair to ensure that participants could maintain their eye level upon the central point of the screen. In addition, the height of the desk was adjusted so that the haptic device was gripped comfortably. They mounted an Oculus Rift Consumer Version 1 HMD (1,080 × 1,200 per eye @90 Hz) and gripped the stylus of a Geomagic Touch device with their dominant hand (Figure 1a) while keeping their thumb on the gray stylus button for submitting responses.

### 3.3. Stimuli and Task

The objective of the task was to compare the size of a visual sphere rendered by the Oculus Rift with a sphere rendered by the haptic device for “feeling” (Figure 1b). A green opaque spherical cursor was rendered superimposed on the haptic proxy point of the Geomagic Touch (Figure 1c). When the task started, a homing position was displayed in the form of a cyan sphere. Participants had to first dock their cursor into the home position; there was no time limit for this step. Upon reaching the home position, both the homing cursor and the user cursor disappeared and an auditory tone was given (c.f. Video figure). Simultaneously, the visual stimulus and the haptic stimulus to be compared were rendered (Visual, rendered in the Oculus Rift, haptic rendered in the Phantom Omni).

The home position was arranged so that upon stimulus onset, the stylus was resting on top of the visual and haptic sphere. i.e., since participants slightly relaxed their arm upon reaching the home position, they automatically rested on the surface of the haptic sphere and were ready to explore.

**Figure 2:**
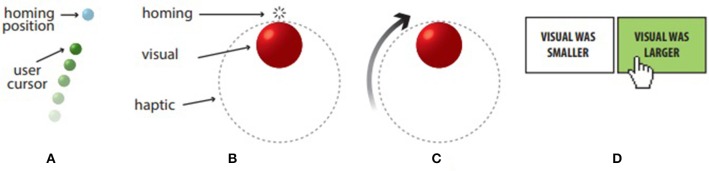
Illustration of the task: **(A)** At the start of a trial, participants were asked to return the cursor to the home position. **(B)** Once the home position was reached, the homing sphere and the user cursor vanished, an auditory tone was given to cue the appearance of the visual sphere and the haptic sphere. **(C)** Users were free to explore the haptic reference. They were instructed to slide the cursor on the surface and loop/explore around the sphere using the haptic device as many times as they could but should not beyond 3 s. **(D)** When the given time period was over, all visual and haptic objects disappeared and a user interface (UI) appeared to show the options for response (“Visual was smaller” or “Visual was larger”). Participants now controlled the X-Y position of a hand cursor that can be moved around to make the “Larger/Smaller” two alternative forced (2-AFC) choice.

Upon stimulus onset, participants were instructed to glide the contact point of the haptic device on the surface of the haptic sphere and complete revolutions around it during a time period of 3 s (Figure 2). After 3 s, the visual and haptic stimuli disappeared and a user interface for making a choice popped up (Figure 1d). Participants then had to respond whether the visual stimulus they saw through the Oculus Rift was larger or smaller than the haptic stimulus they “felt.” Participants controlled a hand cursor using the stylus and pressed the stylus button to submit their response (Figure 2). The UI then disappeared, the cursor was rendered again at the stylus proxy point and the homing position appeared to guide the participant to the home position, in preparation for the next trial.

### 3.4. Variables

The independent variables were haptic reference and *gain*. The haptic reference was controlled at three levels—4, 5, and 6 cm diameter. The gain is the ratio of the diameter of the haptic sphere relative to the visual sphere. A gain of 1.0 means that the red sphere seen through the HMD was identical in diameter to the haptic sphere. A gain of 2.0 means the visual sphere was twice as large as the haptic sphere etc.

We chose seven gain levels of 0.33, 0.55, 0.77, 1.0, 1.22, 1.44, and 1.66. These seven gain levels combined with the three haptic reference levels allow us to conduct a psychometric analysis with two alternative choice (2-AFC) task. We chose those levels by considering that the height of the phantom omni workspace is limited to 12 cm vertically. I.e., 6*cm* × 1.66 = 9.9*cm*. I.e., Had we made the gain or the haptic reference values larger, the resulting rendered sphere in the HMD would be larger than the haptic workspace of the tactile device and therefore impossible to render. Conversely, the smallest haptic reference level was 4 cm, multiplied by the smallest gain (0.33) results in a visual sphere of 1.32 cm diameter. Anything smaller than that would be impossible for participants to glide around and trace using the haptic stylus proxy point.

In total, participants received a test with 3 haptic reference levels × 7 gain values × 10 repetitions per level = 210 trials. All the trials were randomly presented. Before the formal experiment, participants were allowed to familiarize themselves with the device and did 15 practice trials. The experiment lasted ~25 min, including instruction and practice.

## 4. Results

Data from six participants were discarded due to the random responses, which are far beyond the 2.5 standard deviations of the mean, and hence the low quality for the subsequent data fitting. Responses across seven visual gains, under three levels of haptic references, were fitted to the psychometric curve using a logistic function with default parameters (formula 1) (Treutwein and Strasburger, 1999; Wichmann and Hill, 2001).

(1) $$\begin{array}{l} f\left(x\right)=\frac{1}{1+e^{-x}} \end{array}$$

The transitional threshold, that is, the point of subjective equality (PSE) at which the participant was likely to report the visual size was larger than the haptic size, was calculated by estimating 50% of reporting of larger on the fitted curve. The just noticeable difference (JND), an indicator of the sensitivity of size discrimination, was calculated as half of the difference between the lower (25%) and upper (75%) bounds of the thresholds from the psychometric curve.

**Figure 3:**
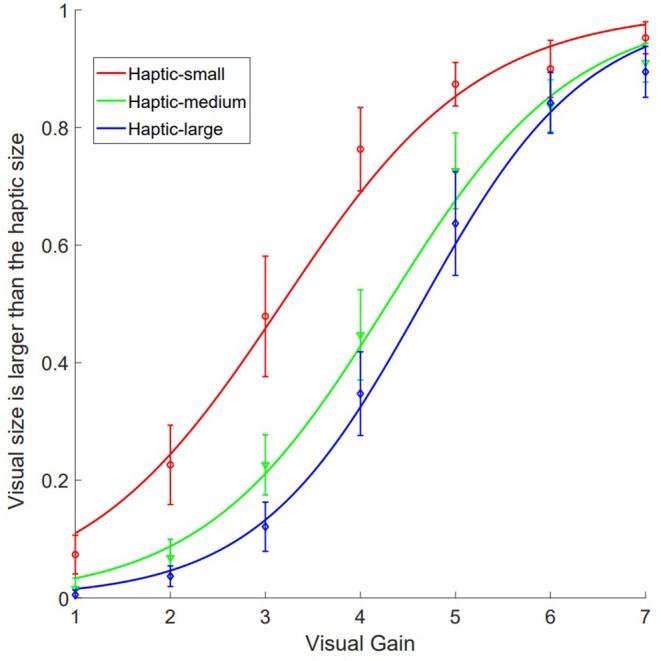
A plot of the psychometric curves.

The mean PSEs for small, medium, and big haptic size references were 3.24 (SE = 0.15), 5.31 (SE = 0.15), and 6.86 (SE = 0.22) (Supplementary Table 1). Repeated measures of ANOVA showed a main effect of the reference haptic size, *F*_(2, 36)_ = 201.47, *p* < 0.001, eta = 0.918. Bonferroni corrected comparisons showed significant differences among the three PSEs, *p* < 0.001. A one-sample *T*-test showed that for the medium reference (size = 5 cm), *t*_(18)_ = 2.018, *p* = 0.059. However, participants over-estimated the visual size in small haptic size reference, *t*_(18)_ = −5.118, *p* < 0.001. They under-estimated the visual sizes for the large haptic size reference, *t*_(18)_ = 3.948, *p* = 0.001. The resulting pattern shows a central tendency effect (Figure 3). The mean PSEs for small, medium, and large references are listed in the following table (Table 1, plot in Figure 3).

**Table 1:** Listing of PSE per haptic sphere reference size.

	**PSE**	**SE**
Small	3.24	0.167
Medium	5.31	0.15
Large	6.86	0.22

For the JNDs, mean JNDs for small, medium, and big haptic size references were 0.94 (SE = 0.11), 1.12 (SE = 0.08), and 1.15 (SE = 0.08), repeated measures of ANOVA showed a main effect of the “reference,” *F*_(2, 36)_ = 3.612, *p* = 0.037, eta = 0.167.

## 5. Discussion

In the present experiment, participants explored and compared the visual and haptic sizes in the peripersonal space with cues from the real world and the VE. The sizes from two modalities were either congruent or conflicting (but with different disparities). The difficulties of the tasks under three tactile size references were controlled well, since the JNDs were statistically the same for the given three conditions.

Results show a dominant functional priority of the visual size perception. In general, a one sample *T*-test showed that the obtained PSEs under three haptic conditions were smaller than the corresponding reference sizes (4, 5, and 6 cm, respectively, *p*s < 0.001). Therefore, participants tended to judge the visual sizes as larger than the haptic sizes, even though they were physically the same. This finding provides novel implications for the design of perceptually realistic visuo-haptic interactions in the peripersonal space.

Moreover, in the context of the general under-estimation perceptions, participants demonstrated a typical central tendency effect: over-estimation for the smaller haptic size but under-estimation for the larger haptic size (Watson, 1957; Thomas et al., 1974; Newlin et al., 1978; Mehrdad and Michael, 2010; Karaminis et al., 2016). Those results could be accounted for in a framework of adaptation level (theory) for haptic size reference during human-computer/machine interaction. Adaptation level theory states that the perceptual discrimination of the comparison properties (here we designated them as visual sizes) with the target properties (haptic sizes), is dependent both on the discrepancies between the two sensory stimuli, and the mean property (of standard stimuli) being introduced. Put in another way, for the given medium size of haptic reference (5 cm in diameter), human observers have consistently demonstrated the central tendency effect and under-estimation of the haptic sizes, compared with the physically same visual sizes. Experiments with a single mean reference are common in the literature. However, in the current study, the setup with two additional references (4 and 6 cm on both ends), has magnified the differences of perceived haptic sizes on the two ends compared to the 5 cm reference condition. Therefore, participants could, to some degree, change their perceptual discriminations by adapting to different levels of the mean properties (small, medium, and large sizes) of the standard stimuli (Helson, 1959, 1964; Eysenck, 1966). This effect has also been shown in other distance perception experiments in VR, in which under-estimation has been found for larger distances, whereas over-estimation has been found for shorter distances.

With that said, there are several potential limitations in this study. We did not collect baseline data, i.e., the judgments of visual sizes and haptic sizes separately across the individuals. Therefore, currently we are not able to implement a cue-combination Bayesian model to quantitatively account for the current findings, as previous studies have done, including Ernst and Banks (2002). For future studies, we could record the grip apertures when participants compared the sizes between the visual and haptic stimuli, to reveal the temporal dynamics when human operators implement goal-directed action in the presence of conflicting perceptual information.

## 6. Conclusion

We studied estimations between visual and haptic sizes when humans actively explore targets and execute certain actions (such as docking based on size information) in peripersonal space and in a virtual environment. Similar to previous studies, we observed spatial dominance of visual size over haptic size (with general over-estimation of visual sizes) when the information is conflicting. Moreover, across the spectrum of haptic sizes for references, human operators demonstrated a typical central tendency effect. We found that our participants over-estimate the visual size when the haptic reference is smaller but under-estimate the visual size when the object haptic reference is larger. This flexibility and adaptivity helps us optimize our actions during human-computer/machine interaction, especially when we primarily rely on different levels of mean sensory properties (including sizes) for perceptual decisions and subsequent action planning and execution.

These results provide interesting implications for the design of perceptually-inspired visuo-haptic interactions in fields related to redirected touching, haptic retargeting due to the changes of visual gain (with respect to haptic properties), as well as passive haptic feedback. For further empirical studies, we plan to simulate more complex scenarios which take into consideration of the combinations of multiple visual/haptic properties, such as size, depth and stiffness of the materials, and examine how the weightings of each dimension evolve during the teleoperation in a VE. In addition, in current settings, we did not investigate spatio-temporal bindings during operation. For potential further studies, we could purposely inject time delay (to mimic transmission latency) of given sensory events during the binding of visual and haptic properties across different visual eccentricities, and discover/measure the efficiency of human performance in VE.

## Data Availability Statement

All datasets generated for this study are included in the article/Supplementary Material.

## Ethics Statement

The studies involving human participants were reviewed and approved by University of Hamburg Informatik Ethics Committee. The patients/participants provided their written informed consent to participate in this study.

## Author's Note

An earlier version of this work has been published in Katzakis et al. (2019).

## Author Contributions

NK designed and programmed the experiment, set-up the apparatus, conducted the experiment, and co-authored the paper. LC conducted the data analysis and co-authored the paper. FS provided the lab space, funding, co-designed the experiment, and co-authored the paper.

### Conflict of Interest

The authors declare that the research was conducted in the absence of any commercial or financial relationships that could be construed as a potential conflict of interest.

## Supplementary Material

Supplementary Table 1 PSEs and JNDs for the experiment.
